# Do you know your r^2^?

**DOI:** 10.5599/admet.888

**Published:** 2020-08-30

**Authors:** Alex Avdeef

**Affiliations:** in-ADME Research, 1732 First Avenue #102, New York, NY 10128 USA

**Keywords:** coefficient of determination, linear correction coefficient, root-mean-square error, linear regression

## Abstract

The prediction of solubility of drugs usually calls on the use of several open-source/commercially-available computer programs in the various calculation steps. Popular statistics to indicate the strength of the prediction model include the coefficient of determination (r^2^), Pearson’s linear correlation coefficient (r_Pearson_), and the root-mean-square error (RMSE), among many others. When a program calculates these statistics, slightly different definitions may be used. This commentary briefly reviews the definitions of three types of r^2^ and RMSE statistics (model validation, bias compensation, and Pearson) and how systematic errors due to shortcomings in solubility prediction models can be differently indicated by the choice of statistical indices. The indices we have employed in recently published papers on the prediction of solubility of druglike molecules were unclear, especially in cases of drugs from ‘beyond the Rule of 5’ chemical space, as simple prediction models showed distinctive ‘bias-tilt’ systematic type scatter.

## Introduction

The ubiquitous coefficient of determination (r^2^) and root-mean-square error (RMSE) are statistics which enumerate the strength of a physical property prediction model [1-4]. Yet their estimated values depend conditionally not only on random errors in the observed data but also on systematic errors generated as a result of limitations in a particular prediction model. When comparing the strength of prediction from different studies based on different models, it is vital to ensure that the same kinds of statistics are invoked.

Here, the commentary confines the discussion to statistics derived by linear regression of scatter plots of log *S*_0_^Obs^ vs*.* log *S*_0_^Calc^ (log *S*_0_ = logarithm of aqueous intrinsic solubility), with observed values treated as dependent variables (y-axis) and calculated values treated as independent variables (x-axis) [3]. Three types of r^2^ and RMSE statistics are considered here: 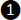 model validation (r^2^_val_, RMSE_val_), 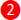 validation with ‘bias’ compensation (r^2^_bias_, RMSE_bias_), and 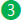 validation with ‘bias-tilt’ compensation, *i.e.*, Pearson’s approach [4] (r^2^_Peason_, RMSE_Pearson_). Whether r^2^ or RMSE is a better statistic to use is beyond the scope of this commentary.

The precise definitions of r^2^ and RMSE are especially pertinent to prediction competitions, for ranking performances consistently. The second ‘Solubility Challenge’ (SC-2) has been described recently [5], modeled after the first competition (SC-1) which took place in 2008 [6]. In SC-2, two test sets of highly-curated aqueous intrinsic solubility data were presented to the computational community to challenge participants to predict the solubility values of the druglike molecules. Concomitant to the SC-2 competition, we also published predictions [7] of the two test sets in SC-2, as well as the test set in SC-1.

Here, we calculated the three types of statistics in order to clarify and put into context the statistics we have employed in our recent studies [7,8], so as to allow consistent comparison of the strengths of our prediction models to those of others [5,6].

## Method

**Figure 1.**
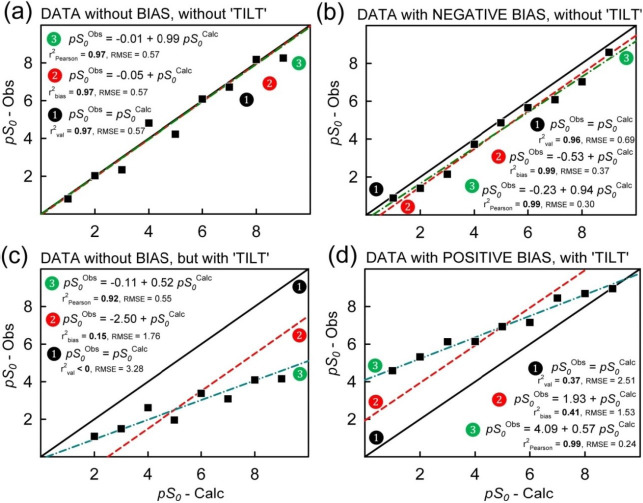
Correlation plots (*pS*_0_ = -log *S*_0_) – three distinct definitions of coefficients of determination (val = model validation, bias = bias compensation, and Pearson), illustrated by simulated data (squares) containing random and systematic errors. The statistics arising from case 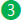 place the prediction in the most favorable light (with RMSE referring to the experimental random error scatter about the green dash-dot curves). Those of case 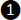 refer to model validation (with RMSE referring to the data scatter about the solid black ‘identity’ diagonal lines). The dashed red lines correspond to the intermediate case 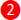.

Figure 1 illustrates the three definitions of the coefficient of determination and the corresponding RMSE, with the aid of simulated data. The ‘observed’ data contain *random errors* of ±(0.24-0.57). The ‘calculated’ data either have no errors (frame a) or have systematic errors (frames b-d). Frame (a) depicts a scatter plot based on a strong prediction model, where the statistics are mainly indications of the random ‘experimental’ errors. The data in frame (b) have a superimposed negative bias, but there is no distortion to the slope in the scatter plot (*i.e.*, no ‘tilt’ to the data trend). Frame (c) has no added bias, but there is a substantial tilt to the data trend (or negative bias without tilt). Frame (d) contains both a positive bias and a tilt added to the random errors (or positive bias without tilt).

The simulated prediction model is assumed to have been ‘trained’ using a large diverse data set. The strength of the prediction can be determined by a randomly-selected smaller set of ‘test’ compounds not used in the training. Three types of statistics may be of interest in the analyzed scatter plot for the test compounds:

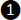
 r^2^_val_ and RMSE_val_ may be used to assess how effectively the training-set derived model predicts the test set (*i.e.*, model validation), as indicated by the dispersion of data about the ‘identity’ line (y = x).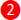 r^2^_bias_ and RMSE_bias_ may be used when the prediction model generates a constant bias (*a*) in the scatter plot, as indicated by the dispersion of data about the unit-slope regression line, displaced from the identity line by the extent of bias (y = *a* + x).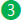 r^2^_Pearson_ and RMSE_Pearson_ Pearson’s statistics [4] are based on regression analysis (y = *a* + *b*x) of a scatter plot showing both bias (intercept, *a*) and ‘tilt’ (slope, *b*). The statistics depend on the dispersions about the (non-unit slope and non-zero intercept) regression line.

The above considerations suggest three constraints for linear regression, y = *a* + *b*x: 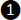 constrained *a* = 0 (no bias) and *b* = 1 (no tilt), 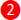 constrained *b* = 1 (no tilt) and determined *a* (bias), 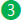 both *a* and *b* determined (without constraints). The statistics which are calculated in these three cases can be quite different, depending on the type and extent of systematic errors.

For case 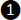, the explicit equations for the two statistics are:


(1)

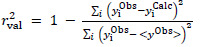




(2)

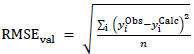



where y = log *S*_0_ and <y^Obs^> is the mean of log *S*_0_ values. The r^2^_val_ in Eq. (1) is often called the ‘coefficient of determination,’ or simply, ‘r-squared.’ According to Eq. (1), if all the calculated log *S*_0_ values match the observed values (‘perfect fit’), then r^2^_val_ = 1. Inappropriate/poor models can lead to r^2^_val_ < 0.

For case 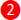 statistics, the bias (*a*) is incorporated into the expressions:


(3)

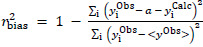




(4)

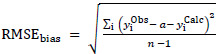



For case 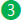 statistics, both the bias (*a*) and the slope factor (*b*) are incorporated into the expressions:


(5)

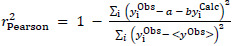




(6)

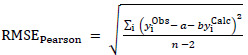



Pearson’s r is more explicitly calculated as [4]


(7)

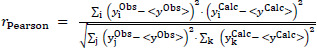



In the absence of systematic errors (Fig. 1a), *it does not matter which of the three definitions is used*. The statistics take on the same values. However, if there is bias (without tilt) in the fit (Fig. 1b), then cases 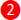 and 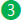 produce comparable statistics, which are ‘better’ than then those of case 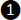. When there is a tilt in the trend or when there is a combined tilt and bias, then the three sets of statistic produce different values, as illustrated in Figures 1c,d. For such cases, r^2^_Pearson_ > r^2^_bias_ > r^2^_val_, while RMSE_Pearson_ < RMSE_bias_ < RMSE_val_. The greater the systematic distortion, the greater the difference between the three sets of metrics. If the source of *random* errors is solely from the data, then RMSE_Pearson_ may be a good indicator of effective measurement errors; RMSE_val_ is the better indicator of overall solubility prediction.

Both Eq. (1) and Eq. (7) are popularly used. But in many publications it is not clear which was actually applied. Also, it may not be readily apparent which r^2^ is calculated in some open-source/commercial programs from the provided documentation. This can lead to some confusion when comparing statistics between independent predictions of solubility coming from different laboratories, using different methods and programs.

## Results and discussion

In our previous publications [7,8] we listed r^2^_bias_ and RMSE_bias_ in our scatter plots *without* the subscript designations, thus inadvertently ascribing them to Eqs. (1) and (2) definitions. In most cases, the differences between the two types of statistics are negligible, but not in all cases. For example, the General Solubility Equation (GSE) and the Abraham Solvation Equation (ABSOLV) models used to predict the solubility of drugs from ‘beyond the Rule of 5’ chemical space showed (*e.g*., Figs. 4b, 5b in Ref. [8]) distinctive bias-tilt type scatter, with different degrees of systematic aberrations introduced by the limitations in the models when applied to such large molecules (similar to what is shown in Fig. 1d here). In contrast, the Random Forest regression (RFR) model (*e.g*., Fig. 13c in Ref. [7] and Fig. 6c in Ref. [8]) was relatively free of such systematic distortions (similar to what is shown in Fig. 1a here), and consequently the three sets of statistics are nearly the same in the RFR examples (*cf*., tables below).

### Sample calculations and possible confusion

In Ref. [7], the GSE was used to predict the 28 intrinsic solubility values taken from the SC-1 competition [6]. Since the GSE requires no ‘training,’ we expected to see some bias and tilt in the resulting scatter plots. Fig. 11b in Ref. [7] shows a log *S*_0_^Obs^ vs. log *S*_0_^Calc^ scatter plot (*cf*., Table 1 below). The statistics listed in that figure are r^2^_bias_ = 0.26 and RMSE_bias_ = 1.23.

We used SigmaPlot to construct publication-quality figures. In the accompanying statistics calculation, the bias was determined by fitting the function: log *S*_0_^Obs^ = *a* + *b*log *S*_0_^cal^, where the *b* regression coefficient was constrained to be 1.0, so the determined bias = *a*. In the above Fig. 11b example, the calculated bias = -0.61 log unit. SigmaPlot calculated the values ‘Rsqr’ = 0.26 and ‘Standard Error of Estimate’ = 1.23, which we listed in the plot. This is consistent with the calculations of Eqs. (3) and (4).

However, Eqs. (1) and (2) produce r^2^_val_ = 0.07 and RMSE_val_ = 1.34.

Furthermore, for the same example, the open-source default cor(x,y) function [9] calculated ‘r-squared’ = 0.45 and the sample script function defined by Walters [2] calculated ‘rmsError’ = 1.07. This is consistent with the calculations of Eqs. (5) and (6) – Pearson’s equations.

So, the three ‘r-squared’ statistics were calculated as 0.07, 0.26, and 0.45 and the corresponding ‘RMSE’ values were 1.34, 1.23, and 1.07, respectively. This can be confusing when comparing prediction models. It’s not that any of these values is wrong – it’s just that different equations/assumption are used/implied. Generally, the appropriate definition of the coefficient of determination is according to Eq. (1) and the RMSE is according to Eq. (2), since these focus on the actual strength of the model in linking prediction to measurement.

### Recalculation of the statistics for our previous studies

Tables 1 and 2 list three types of ‘r-squared’ and root-mean-square errors for the scatter plots in Refs. [7] and [8]. In these two studies, we used the bias-compensated statistics originating from the SigmaPlot calculation, but inadvertently ascribed them to Eqs. (1) and (2). As can be seen in cases where the bias is negligible, the three sets of statistics are nearly the same (*e.g.*, Fig. 8 [7] or Fig. 6 [8] RFR results in Tables 1, 2). In many of the scatter plots, the differences between the different sets of statistics are very small.

## Conclusion

Statistics from ready-made programs may be easily verified (*e.g*., spreadsheet calculation using Eqs. (1)-(6)), so that the intended values are reported. The expanded calculations of statistics (Tables 1 and 2) applied for our recent prediction studies [7,8] should now allow for valid comparisons between the strength of our predictions of solubility to those reported by others: *e.g*., in ‘Solubility Challenges’ SC-2 [5] and SC-1 [6].

## Figures and Tables

**Table 1.** Recalculated statistics for the scatter plots in Ref. [7]

Type ^a^	Fig. in Ref. [7]	r^2^_Pearson_ Eq. (5)	r^2^_bias_ Eq. (3) ^b^	r^2^_val_ Eq. (1)	RMSE_Pearson_ Eq. (6)	RMSE_bias_ Eq. (4) ^b^	RMSE_val_ Eq. (2)	bias Eq. (3)
GSE, acids	**6a**	0.62	0.61	0.58	1.21	1.24	1.27	-0.29
GSE, bases	**6b**	0.60	0.57	0.56	1.16	1.21	1.21	-0.14
GSE, neutrals	**6c**	0.61	0.54	0.54	1.05	1.15	1.18	-0.30
GSE, zwitterions	**6d**	0.24	0.07	0.02	1.38	1.54	1.57	0.34
ABSOLV, acids	**7a**	0.66	0.66	0.65	1.14	1.15	1.16	-0.15
ABSOLV, bases	**7b**	0.64	0.64	0.62	1.10	1.10	1.13	-0.28
ABSOLV, neutrals	**7c**	0.61	0.61	0.61	1.05	1.05	1.05	-0.11
ABSOLV, zwitterions	**7d**	0.68	0.68	0.67	0.90	0.90	0.92	-0.20
RFR	**8a**	0.98	0.98	0.98	0.28	0.28	0.28	0.00
RFR	**8b**	0.90	0.89	0.90	0.60	0.60	0.60	-0.02
RFR, zwitterions	**8b -**inset	0.91	0.91	0.91	0.45	0.45	0.45	0.01
GSE, Test Set 1	**11a**	0.78	0.78	0.73	0.97	0.97	1.01	-0.41
GSE, Test Set 2	**11b**	0.45	0.26	0.07	1.07	1.23	1.34	-0.61
GSE, Test Set 3	**11c**	0.46	0.26	0.20	0.94	1.10	1.13	-0.31
GSE, Test Set 4	**11d**	0.69	0.69	0.68	1.23	1.24	1.25	-0.08
ABSOLV, Test Set 1	**12a**	0.77	0.69	0.58	0.98	1.15	1.27	-0.65
ABSOLV, Test Set 2	**12b**	0.55	0.55	0.35	0.98	0.98	1.13	-0.62
ABSOLV, Test Set 3	**12c**	0.47	0.36	0.26	0.94	1.02	1.10	-0.41
ABSOLV, Test Set 4	**12d**	0.72	0.72	0.70	1.18	1.18	1.18	-0.29
RFR, Test Set 1	**13a**	0.90	0.83	0.82	0.66	0.84	0.83	-0.23
RFR, Test Set 2	**13b**	0.66	0.66	**0.57**	**0.85**	0.85	0.92	-0.41
RFR, Test Set 3	**13c**	0.66	0.66	0.64	0.74	0.75	0.76	-0.18
RFR, Test Set 4	**13d**	0.82	0.77	0.71	0.95	1.05	1.15	-0.54
GSE, Test Set 1	**14**	0.91	0.90	0.89	0.62	0.66	0.66	0.02

^a^ GSE = General Solubility Equation; ABSOLV = Abraham Solvation Equation; RFR = Random Forest regression.

^b^ Statistics reported in Ref. [7].

**Table 2.** Recalculated statistics for the scatter plots in Ref. [8]

Type	Fig. in Ref. [8]	r^2^_Pearson_ Eq. (5)	r^2^_bias_ Eq. (3) ^a^	r^2^_val_ Eq. (1)	RMSE_Pearson_ Eq. (6)	RMSE_bias_ Eq. (4) ^a^	RMSE_val_ Eq. (2)	bias Eq. (3)
GSE, small molecules	**4a**	0.62	0.59	0.57	1.17	1.21	1.23	-0.22
GSE, large molecules	**4b**	0.48	-3.8	-3.82	1.00	3.05	2.95	0.16
GSE, modified	**4c**	0.48	0.34	0.33	1.00	1.13	1.1	0.04
ABSOLV, small molecules	**5a**	0.67	0.67	0.66	1.08	1.08	1.1	-0.2
ABSOLV, large molecules	**5b**	0.13	-1.39	-5.24	1.30	2.15	3.36	-2.64
ABSOLV, modified	**5c**	0.48	-0.91	2.07	1.01	1.92	2.07	0.92
RFR, training set	**6a**	0.98	0.98	0.98	0.26	0.27	0.27	0.00
RFR, internal validation	**6b**	0.89	0.89	0.89	0.64	0.64	0.64	0.02
RFR, large molecules	**6c**	0.45	0.42	0.37	1.03	1.06	1.07	0.30

^a^ Statistics reported in Ref. [8].
